# Currently applied sonication protocols for the detection of implant-associated infections in orthopaedics: a scoping review

**DOI:** 10.1128/jcm.00495-26

**Published:** 2026-06-22

**Authors:** Nader Maai, Florian A. Frank, Martin Clauss, Sonostandard Research Group, Nader Maai, Richard A. Kuehl, Mario Morgenstern, Peter M. Keller

**Affiliations:** 1 AO Research Institute Davos 161930 , Davos, Switzerland; 2 Department of Orthopaedics and Trauma Surgery, University Hospital Basel 30262 , Basel, Switzerland; 3 Centre for Musculoskeletal Infections (ZMSI), University Hospital Basel 30262 , Basel, Switzerland; 4 Centre for Infectious Diseases Diagnostics, University Hospital Basel 30262 , Basel, Switzerland; 5 Clinical Bacteriology / Mycology, University Hospital Basel 30262 , Basel, Switzerland; 6 Department of Infectious Diseases, University Hospital Basel 30262 , Basel, Switzerland; Cleveland Clinic, Cleveland, Ohio, USA

**Keywords:** sonication, periprosthetic joint infection, fracture related infection, sonication fluid culture, diagnostic yield, CFU threshold

## Abstract

Implant-associated infections, including periprosthetic joint infections (PJI) and fracture-related infections (FRI), are severe complications following orthopedic surgery. Conventional tissue cultures often yield false-negative results, especially after antibiotic initiation or in chronic biofilm-associated infections. Sonication of explanted implants enhances bacterial recovery, but heterogeneous protocols limit comparability. This scoping review maps sonication protocols currently reported in the literature. A comprehensive literature search across three databases (PubMed, Embase, and Scopus) was performed. Clinical studies that reported on sonication protocols used in the microbiological workup of PJIs and FRIs published from 2007 to October 2025 were eligible for inclusion. Eligible studies were screened for the following information: container size and material and fluids, sonication device and settings, pre- and post-sonication handling, culture conditions, reporting format, and/or diagnostic thresholds. Data were extracted and synthesized descriptively. After duplicate removal, 312 records were screened, and 146 studies were included. Reporting was incomplete for several key parameters. Container material was not specified in 43.2% of studies, container type in 30.8%, and fluid type in 31.0%. Among reported protocols, hard plastic containers (54.8%) and Ringer’s lactate or saline (40.4%) were most frequently used, with fluid volumes ranging from 50 to 800 mL. Sonication frequency was reported in 50.7% of studies, most commonly 40 kHz (28.8%). Sonication time was not reported in 34.2%, with reported durations ranging from 1 to 10 min and most frequent duration as 5 min (30.1%). Vortexing and centrifugation were applied in 55.5% and 45.9% of studies, respectively. CFU thresholds varied widely, ranging from any growth to ≥50 CFU/mL or per plate. Time-to-culture and storage conditions were rarely reported. Sonication protocols vary greatly across almost all parameters, with detailed descriptions often lacking, and certain variables rarely reported. Standardizing these protocols is essential to ensure reproducibility and to establish clinically reliable diagnostic cut-offs.

**IMPORTANCE:**

Fracture-related infections (FRI) and periprosthetic joint infections (PJI) are serious complications associated with substantial morbidity, repeated surgeries, and a high healthcare burden. Accurate and early pathogen detection is critical for guiding targeted antimicrobial therapy and improving treatment outcomes. Sonication of explanted implants has emerged as a valuable diagnostic tool by disrupting biofilms and enhancing bacterial recovery, particularly in antibiotic-exposed patients. However, sonication protocols vary widely across studies, including differences in containers, fluids, ultrasound settings, processing steps, and colony-forming unit thresholds, with inconsistent reporting further compounding the issue. This variability directly affects diagnostic performance, complicates the distinction between true infection and contamination, and limits inter-laboratory comparability. By systematically mapping this heterogeneity across 146 studies, this scoping review provides a foundation for developing standardized protocols and consensus definitions to improve diagnostic reliability and patient care.

## INTRODUCTION

Implant-associated bone and joint infections such as periprosthetic joint infections (PJIs) and fracture-related infections (FRI) represent serious complications imposing increased morbidity, repeated surgical interventions, prolonged hospitalization, and substantial socioeconomic and healthcare costs to the patient and healthcare systems (1–3).

Despite recent advances in definition and diagnostics, up to 16%–30% of PJIs remain culture-negative, particularly after prior antibiotic exposure (4–7). A central mechanism is bacterial biofilm formation on implant surfaces, which enables pathogen persistence and immune evasion. Biofilms are composed of complex microbial consortia embedded in extracellular polymeric substances that protect against host defenses and antimicrobial therapy (8), establishing a protected niche on the implant surface and contributing to false-negative results in conventional cultures of the surrounding tissue (9).

The benefit of sonication was shown in the seminal clinical study by Trampuz et al. in 2007 (10). The technique uses ultrasonic energy to disrupt biofilms and release adherent bacteria into a suspension for culture. It is a physical process distinct from ultrasound imaging. The findings of Trampuz et al. which demonstrated a markedly higher diagnostic yield compared with tissue culture were later corroborated by others (11). Subsequent studies refined the method by adjusting sonication time, fluid volume, and incorporating steps such as centrifugation, vortexing, and molecular assays (12, 13). The main advantages of sonication are higher diagnostic yield, fewer false negatives, and improved detection of slow-growing pathogens such as *Cutibacterium acnes* (14). By 2024, routine sonication was recommended for all revision arthroplasties, given its superior pathogen recovery when conventional techniques remain inconclusive (15). Integration of molecular methods with sonicate fluid further increases sensitivity, especially in antibiotic-treated patients in which cultures frequently fail (16).

Protocol heterogeneity remains a key barrier to standardization, with marked differences in ultrasound frequency, agitation time, and colony-forming unit thresholds for infection (13). These inconsistencies limit comparability across studies and hinder broad clinical implementation, emphasizing the need for optimized, uniform methods to reliably distinguish aseptic failure from true infection, especially in antibiotic-exposed patients (6). As outcomes are highly protocol-dependent, critical variables include fluid type and volume, ultrasound frequency and duration, as well as subsequent vortexing, centrifugation, and/or culture conditions.

Comparative studies report sensitivities of 75%–92.5% for sonication fluid cultures; however, diagnostic performance varies across studies and depends on factors such as the number and quality of tissue specimens and methodological differences (2, 17, 18). Sonication should, therefore, be considered an adjunct to conventional tissue cultures rather than a standalone diagnostic method. Use of automated liquid culture systems such as BD BACTEC further improves pathogen recovery (19). The most frequently identified organisms are coagulase-negative staphylococci and *Staphylococcus aureus*. Low-virulence bacteria, such as anaerobes and *C. acnes*, are also detected more reliably than with conventional methods (5). Epidemiologically, staphylococci account for 60%–70% of PJIs, *C. acnes* and anaerobes for 10%–15%, and Gram-negative bacilli or fungi for 5%–10%, often in culture-negative or polymicrobial cases (20). Molecular adjuncts, including multiplex and real-time PCR as well as broad-range 16S rRNA analysis, can raise sensitivity to nearly 96%, enabling pathogen detection even in culture-negative cases (6, 21).

The aim of this scoping review is to map the currently available sonication protocols for PJI and FRI.

## MATERIALS AND METHODS

The scoping review was conducted under the Preferred Reporting Items for Systematic Reviews and Meta-Analyses Extension for Scoping Reviews (PRISMA-ScR) guidelines (22).

### Database search protocol

A search protocol was developed incorporating the search strategy, keywords, and inclusion and exclusion criteria. The authors reviewed and refined the search strategy to ensure comprehensiveness and appropriateness of the search terms.

A systematic literature search was performed on 15th of October 2025 across three electronic databases: PubMed, Embase, and Scopus. The search strategy combined Medical Subject Headings (MeSH) and free-text keywords related to sonication and implant-associated infections (Table S1). The core search terms consisted of “sonication AND periprosthetic joint infection,” “sonication AND prosthetic joint infection,” “sonication AND PJI,” “sonication AND fracture-related infection,” and “sonication AND FRI.” Boolean operators were applied to capture all studies reporting the use of sonication in the microbiological workup of PJI or FRI. The search covered publications from January 2007 up to October 2025. All clinical studies were included, and laboratory studies were excluded. All retrieved records were exported into EndNote (Version 21, Clarivate, USA) for deduplication and reference management.

### Study eligibility, selection, and data extraction

Following deduplication, two independent reviewers screened the titles and abstracts to identify studies that reported any aspect of a sonication protocol. Full texts were retrieved for all eligible articles, and studies not available in English or German, abstract-only publications, reviews, laboratory-only experiments without clinical relevance, and studies unrelated to the research question were excluded. Studies that merely cited another sonication protocol without providing methodological details were marked as insufficiently reproducible and excluded from data synthesis. Disagreements between the reviewers were resolved through consultation, consensus, or recruiting a third reviewer to break the tie.

Two independent reviewers screened the selected studies and extracted data using a structured spreadsheet (Microsoft Excel, Microsoft Corp., USA). Extracted variables included container type and material, liquid type and volume used during sonication, sonication device, frequency, power, and duration, as well as pre- and post-sonication handling steps such as vortexing, manual shaking, or centrifugation. Additional information included the tested fluid volume, culture media and incubation conditions, CFU-reporting format (CFU/mL, CFU/plate, or total CFU), diagnostic thresholds, time to culture, and storage conditions. CFU threshold was recorded where available and could vary depending on causative pathogen prior to antimicrobial exposure and variability in diagnostic frameworks, for example, those proposed by the European Bone and Joint Infection Society (EBJIS).

When studies omitted one or more of these parameters, this was documented as missing information. Disagreements were resolved through consultation, consensus, or recruiting a third reviewer to break the tie.

A PRISMA flow diagram (attached in the Supplemental material) illustrates the identification, screening, and inclusion process, detailing the number of records retrieved, duplicates removed, full texts assessed, and final studies included in the review.

## RESULTS

As shown in Fig. S1, the initial search yielded 698 records. After removing 386 duplicates, 312 records remained for title and abstract screening. After screening, 51 irrelevant and unpublished studies were removed, and the remaining 260 articles were assessed in detail. Sixteen records could not be retrieved due to the lack of full text or because they were abstract-only publications. A further six articles were excluded for being written in languages other than English or German. In addition, 42 review articles, 20 purely laboratory-based studies, and 31 studies unrelated to the research question were excluded. Ultimately, 146 studies met the eligibility criteria and were included for data extraction.

Across studies, performance metrics were reported. Sensitivity in sonication fluid culture (SFC) widely varied across studies ranging from 47% (23) to as high as 96% (6), generally outperforming tissue cultures in chronic PJI and in patients treated with antibiotics. Specificity was generally high across studies >85% though some studies reported lower specificity depending on CFU thresholds. Sonication combined with vortexing, centrifugation, or enrichment in blood cultures appeared to improve the detection of pathogens, including low-virulence and anaerobic organisms. PCR, multiplex PCR, and metagenomic sequencing applied to sonication fluid demonstrated higher sensitivity than culture alone, especially in antibiotic-exposed patients.

### Containers and fluids

Details on container types and materials as well as fluid types are summarized in Table 1. Fluid temperature was never reported, while container sterility was inconsistently reported, despite their potential impact on sample contamination and viability (24, 25).

**TABLE 1:** Container properties and fluids for sonication

Containers and fluids	*n* (number of studies)	Percentage
Container material		
Plastic containers	80	54.8
Metal or hard autoclaved body	3	2.1
No information provided	63	43.2
Container type		
Hard container	98	67.1
Bag	2	1.4
Hard container or bag	1	0.7
No information provided	45	31.0
Amount of fluid added		
50–800 mL	47	32.2
Enough to submerge the device	15	10.3
Enough to cover 90% of the device	11	7.5
Enough to cover 80% of the device	2	1.4
No information provided	71	48.6
Fluid type		
Ringer’s (solution, lactate)	59	40.4
Saline/0.9% Sodium chloride/PBS	32	21.9
Ampuwa (Fresenius Kabi, sterile water)	3	2.1
No information provided	52	35.6

#### Container types and materials

Out of the 146 studies reviewed, 80 studies specified (54.8%) using plastic containers, mainly consisting of polypropylene. Containers were labeled as sterile, single-use or reusable, lock and lock airtight plastic containers. Only one study indicated using metal (26) and two others hard autoclavable cylinder body containers (6). In terms of the type of container (bag or hard container), 98 studies (67.1%) specified that the containers were hard containers with a cylindric body, sealed, sealable or screw top, autoclavable, and having an airtight seal. Two studies indicated using a bag (27, 28). One study revealed using either hard containers or bags (29). Notably, a significant number of studies (*n* = 45, 30.8%) did not specify the material of the container used.

#### Amount and type of liquid added

There was considerable variation in the type and volume of fluid used for sonication (Table 1). In most studies, the volume depended on implant size, with smaller devices receiving lower volumes (approximately 50–200 mL) and larger implants such as hip and knee components requiring higher volumes (up to 400–500 mL) (10, 30, 31). Only a portion of studies reported exact volumes, whereas many simply described adding fluid sufficient to submerge the implant or did not specify the volume at all. Ringer’s lactate and sterile saline were the most frequently reported fluids although several studies used alternative solutions. Notably, a substantial proportion of studies did not report the type or volume of fluid used (32, 33).

### Sonication devices and settings

The BactoSonic BS 14.2 (BANDELIN GmbH, Berlin, Germany) was the most frequently used sonication device, with various Branson models representing the next most common group. Other systems, such as Aquasonic baths, generic ultrasound baths, Woxing units, CQ-200B-DST, VS-TP24 ultrasonic devices, portable handheld units, and Sonic-6, were reported only rarely, each in very few studies.

Sonication frequency parameters also showed variability (Table 2). Most protocols operated at or around 40 kHz, including those that reported fixed 40 kHz settings as well as small tolerances (e.g., 40 ± 5 kHz or 40 ± 2 kHz). One study reported a sonication frequency setting of 67 kHz (34). Four studies applied frequencies below 40 kHz (30 kHz [35], 28 ± 2 kHz [36], 25 ± 5 kHz [26]). One study reported a sonication frequency of 40 Hz, which is by definition not ultrasound (37). The detailed distribution of devices and frequency settings is summarized in Table 2.

**TABLE 2:** Sonication devices and settings across studies

Sonication device and settings	*n* (no. of studies)	Percentage
Sonication device		
BactoSonic (BANDELIN GmbH, Berlin, Germany)	49	33.6
Branson (Emerson Electric, Danbury, CT, USA)	19	13.0
Aquasonic (VWR, Avantor, Radnor, Pennsylvania, USA)	3	2.1
Ultrasound bath (VWR, Avantor, Radnor, Pennsylvania, USA)	4	2.7
Woxing (Wuxi Woxin Instrument, Wuxi, Jiangsu, China)	2	1.4
CQ-200B-DST (Shanghai Yuejin Medical Equipment Co, Shanghai, China)	2	1.4
VS-TP24 Ultrasonic cleaner (Wuxi Woxin Instrument, Wuxi, Jiangsu, China)	1	0.7
Portable handheld ultrasonic Shanghai Weimi Ultrasonic Co., Ltd., Shanghai, China)	1	0.7
Sonic-6 (SMS) (Polsonic, Warsaw, Poland)	1	0.7
DH-WUC D10H (Daihan Scientific Co., Ltd, Wonju, South Korea)	1	0.7
No information provided	63	43.2
Sonication settings (kHz)		
67 kHz	1	0.7
40 kHz	42	28.8
40 ± 2 kHz	19	13.0
40 ± 5 kHz	8	5.5
30 kHz	1	0.7
28 ± 2 kHz	1	0.7
25 ± 5 kHz	1	0.7
40 Hz	1	0.7
No information provided	72	49.3

### Pre and post-sonication handling

Pre- and post-sonication handling protocols revealed notable variations in timing, duration, type, and mode of handling (centrifugation, handshaking, and vortexing) (Table 3).

**TABLE 3:** Pre- and post-sonication handling variability

Pre- and post-sonication handling	*n* (number of studies)	Percentage
Sonication time		
1 min	38	26.0
2 min	1	0.7
3 min	5	3.4
5 min	44	30.1
7 min	5	3.4
10 min	3	2.1
No information provided	50	34.2
Vortex step		
*Yes*	81	55.5
Before	8	5.5
After	9	6.2
Both	41	28.1
Not specified	23	15.8
No information provided	54	37.0
** ** *No*	11	7.5
Manual handshaking		
*Yes*	11	7.5
Before	1	0.7
Before and after	7	4.8
Not specified	3	2.1
No information provided	54	37.0
*No*	81	55.5
Centrifugation		
*Yes*	67	45.9
Before plating (RPM 3,000–4,000; 5–15 min**)**	11	7.5
For further testing, e.g., PCR (RPM 1,000–3,000; 10 min**)**	56	38.4
No information provided	46	31.5
*No*	33	22.6
PCR performed		
*Yes*		
Broad-range 16S rRNA PCR	15	10.3
Multiplex PCR	20	13.7
Lab-developed PCR	21	14.4
Metagenomic sequencing	7	4.7
*No*	91	62.3
Volume of liquid test		
0.1 mL	40	27.4
0.5 mL	17	11.6
1 mL	22	15.1
10 mL	18	12.3
100 mL	12	8.2
No information provided	37	25.3
Automated testing		
BD BACTEC	8	5.5
No information provided	138	94.5

#### Sonication time

Sonication duration varied across the analyzed protocols, most commonly ranging from 1–5 min (10, 14, 38), though some studies used longer sonication periods, extending up to 7 or even 10 min (2, 25, 36, 39). Notably 24 papers (16.4%) referred to Trampuz et al. without providing further details. Piper et al. (40), Monsen et al. (25), and Estaban et al. (41) were referred to by three papers each without further detail given.

#### Vortexing

Vortexing was used in more than half of the studies either before, after, or before and after sonication (13, 42, 43). Only 11 (*n* = 11, 7.5%) studies specified that they did not employ vortexing.

#### Manual handshaking

Manual handshaking was far less often applied in the studies reviewed, with only 11 studies (7.5%) indicating the use of such a procedure (2, 23, 30, 44, 45). One study (15) applied hand shaking before sonication, while seven studies (2, 23, 30, 44–46) reported the procedure before and after sonication. None of the studies indicated hand shaking solely after sonication. Many studies (*n* = 81, 55.5%) indicated not having used handshaking procedure at any point before or after sonication.

#### Centrifugation

Centrifugation was a common post-sonication processing step to concentrate bacteria or reduce debris though it was not universally applied across protocols. Almost 50% of the protocols incorporated centrifugation (*n* = 67, 45.9%). Most of these studies used centrifugation to concentrate the fluid for further PCR testing (*n* = 56, 38.4%). In 10 studies (6.8%), centrifugation was used to further concentrate the sonication fluid prior to plating (13, 47–50). Centrifugation parameters across studies varied substantially ranging from 250 to 14,000 rpm (approximately 1,490–15,000 × *g*) with durations between 5 and 30 min. Most protocols exhibited moderate centrifugation conditions, which is between 2,500 and 4,000 rpm (approximately 3,000–4,200 × *g*) for 5–15 min. The centrifugation was frequently performed at 4°C. The findings showed that centrifugation in some protocols was occasionally in multiple sequential steps further concentrating sonication fluid prior to PCR or other downstream molecular analyses. Thirty-three studies (22.6%) did not employ centrifugation, while 46 (31.5%) did not provide any information.

### Culture conditions and CFU thresholds

Details on cultures and diagnostic thresholds are summarized in Table 4.

**TABLE 4:** Culture conditions and CFU thresholds

Culture conditions and CFU threshold	*n* (number of studies)	Percentage
Culture medium		
Blood agars	99	67.8
Chocolate agar	41	28.1
MacConkey agar	26	17.8
Thioglycolate broth	33	22.6
Sabouraud agar	14	9.6
Brucella agar	9	6.2
Brain heart infusion	5	3.4
Cooked meat broth	5	3.4
Blood culture bottles	8	5.5
Unspecified aerobic and anaerobic	7	4.8
No information	31	21.2
CFU threshold		
CFU/mL		
≥50 CFU/ mL	42	28.8
** **≥30 CFU/ mL	1	0.7
≥20 CFU/ mL	3	2.1
≥2 CFU/mL	11	7.5
≥1 CFU/10 mL	2	1.4
CFU/plate		
≥50 CFU/plate	3	2.1
≥20 CFU/plate	2	1.4
≥5 CFU/plate	9	6.2
≥1 CFU/plate	11	7.5
No information provided	64	43.8
Delay in processing		
Within 1 h	7	4.8
Within 6 h	22	15.1
Within 24 h	5	3.4
Within 48 h	3	2.1
Immediately	15	10.3
No information provided	93	63.7
Temperature of storage		
4°C	4	2.8
37°C for 30 min	1	0.7
Room temperature	6	4.1
No information provided	138	94.5

#### Agar used

Out of the 146 studies reviewed, 99 (67.8%) used blood agars as the primary culture medium. This likely reflects the widespread routine use of blood agar for bacterial recovery in clinical biology although differences in culture yielding depending on specimen quality and methodological variability were observed across studies. Common additional cultures used in the studies included chocolate agar (*n* = 41, 28.1%), thioglycolate broth as an enrichment medium (*n* = 33, 22.6%), and MacConkey agar for Gram-negative organisms (*n* = 26, 17.8%). Other less preferred culture media included Sabouraud dextrose sugar (*n* = 14, 9.6%), brucella blood agar (*n* = 9, 6.2%), blood culture bottles (*n* = 8, 5.5%), brain heart infusion (*n* = 5, 3.4%), and cooked meat broth (*n* = 5, 3.4%). Seven studies (4.8%) used aerobic and anaerobic media without further specification. Five studies (3.4%) reported incorporating automated blood culture systems like BD BACTEC peds plus/F with standard aerobic or anaerobic plates and enrichments to maximize bacterial recovery (51, 52).

#### Amount of liquid tested

The volume of liquid extracted from the sonication fluid for further testing varied considerably across studies reviewed, with results showing a preference for smaller liquid volumes. Forty studies (27.4%) reported using 0.1 mL, 17 studies (11.6%) used 0.5 mL, 22 studies (15.1%) 1 mL, 18 studies (12.3%) 10 mL, and 12 studies (8.2%) 100 mL.

#### CFU threshold

Results were reported as CFU per milliliter, per plate, or as total CFU. Considerable heterogeneity existed in the thresholds used to define a positive culture. Of the 146 studies reviewed, 42 (28.8%) applied a cut-off of ≥50 CFU/mL, whereas 3 studies used a threshold of 50 CFU/plate to define a positive result. Additional details on the applied thresholds are provided in Table 4.

### Time to testing and storage temperature

Time to testing and storage in sonication varied across studies, with multiple studies reporting processing samples immediately or ideally within 4 to 6 h (13, 53). However, immediate processing was not feasible in some settings, and the samples were stored at 4°C without liquid for up to 24 h, while refrigerated samples were stored for even longer durations of up to 7 days (53). Fifteen studies reported immediate testing after surgery (10.3%), others delayed processing for an hour (*n* = 7, 4.8%), a maximum of 6 h (*n* = 22, 15.1%), the same day (*n* = 5, 3.4%), or until 48 h (*n* = 3, 2.1%). The temperature of storage was inconsistent across studies, with four studies storing the sample(s) at 4°C, one study at 37°C for 30 min and six studies at room temperature (Table 4).

## DISCUSSION

This review highlights the substantial methodological heterogeneity in published protocols for implant sonication in the context of PJI and FRI. This variability was observed through all parameters in sonication devices and settings, container materials, liquid type and volume, handling before and after sonication, and culture conditions. These differences complicate cross-study comparison and impede the definition of universally applicable diagnostic thresholds.

Fluid type and volume influence bacterial recovery but reported volumes range from 50 mL to 800 mL depending on implant size (10, 30, 31), often based on visual estimation rather than standardized criteria. Larger volumes dilute bacterial suspensions, particularly affecting the detection of low-virulence organisms (54). Of interest, slightly acidic solutions, such as 0.9% NaCl or Ringer’s lactate, can hinder growth for sensitive organisms, whereas hypotonic fluids, such as Ringer’s lactate, can cause lysis in fragile bacteria (32, 33). Ringer’s or saline were most commonly used (10, 21, 55) though PBS may offer advantages in buffering and maintaining bacterial viability (32, 33). Fluid temperature and container sterility were rarely reported despite their potential impact (24, 25). Sonication parameters were also inconsistent. Frequencies around 40 kHz at ~0.22 W/cm² appear to be optimal for biofilm disruption while maintaining bacterial viability (56, 57). Lower or misreported frequencies can yield less reliable results, as illustrated by Fang et al. (37). Sonication duration varied between 1 and 10 min (2, 10, 14, 36, 38), with longer times improving biofilm disruption but potentially reducing viability of organisms (25, 39).

Handling steps such as vortexing, shaking, or centrifugation were variably applied. Vortexing before and after sonication enhanced bacterial release due to biofilm disruption (42). Kobayashi et al. and Silva et al. reported that vortex is a simple and safe way to dislodge bacteria from the implant surfaces before sonication and resuspend them after sonication (13, 42). The method carries the risk of cross-contamination by sample splashing or lysis (13). Manual shaking was less frequently used (2, 23, 30, 44, 45). Centrifugation improved sensitivity, but protocols varied widely: 3,000–4,000 rpm for 5–15 min (47–49) with Silva et al. recommending 4,200–4,500 × *g* for 10–15 min (13). Centrifugation parameters, including relative centrifugal force and duration, may influence bacterial sedimentation and recovery, with centrifugation commonly used to concentrate sonication fluid and improve microbial detection (41, 58).

Culture conditions and thresholds also varied. While nowadays a threshold of 50 CFU/mL is widely used, the original cut-off proposed by Trampuz et al. in their sentinel study was ≥5 CFU/plate corresponding to 10 CFU/mL. Some studies lowered thresholds to ≥20 CFU/mL or even any growth for high-virulence or antibiotic-treated cases (10, 13). Such adjustments may maximize sensitivity, but risk reducing specificity due to overreporting of contaminants. Extended incubation up to 2 weeks increased not only detection (59) but also contamination rates. Automated anaerobic incubation improved sensitivity, particularly in antibiotic-treated patients (60, 61).

Processing time and storage differed considerably, ranging from immediate handling to delays of up to 48 h. While several studies recommended processing within 4–6 h (13, 53), some stored samples refrigerated for up to 7 days (53). Delayed processing may reduce bacterial load, especially of fastidious organisms, although its impact on culture positivity remains undetermined. Delayed processing may also hypothetically increase the risk of contaminants (53).

It must be noted that there are certain limitations to standardization: Adjusting sonication volumes to implant type and size would be theoretically preferable, but implementing a standardized protocol that accommodates such variability is logistically challenging. Moreover, laboratories accredited under ISO 15,189 must revalidate any methodological change, adding significant workload. Laboratories operating under CLIA regulations (administered by CMS) and/or accredited by the College of American Pathologists (CAP) would similarly be required to perform validation studies when implementing or modifying such procedures. Finally, sonication is not an *in vitro* diagnostic (IVD)-certified diagnostic workflow, which limits broad clinical adoption. In addition, there is currently no external quality assurance (EQA) provider (such as UK NEQAS) offering ring trials for sonication, further hindering standardized external quality control.

There were limitations in this review that should be acknowledged. Substantial methodological heterogeneity was observed across the studies evaluating sonication protocols. Differences were noted regarding sonication duration, frequency and power settings, vortexing procedures, centrifugation, and microbiological processes such as culture media and incubation conditions. Different studies employed different diagnostic reference standards in the assessment of sonication protocol performance. These variations limit direct comparisons of protocols and preclude the identification of an optimal protocol. Highlighting these variables was a key objective of this scoping review and demonstrates the lack of standardized sonication and microbiological procedures. Some research groups contributed multiple studies over time. These were included individually, as protocol details or study characteristics differed between publications, allowing a more comprehensive mapping of methodological variability.

We did not perform a formal comparison of diagnostic performance across protocols. The aim of this scoping review was to map currently available sonication protocols and identify areas requiring standardization rather than to rank protocols by diagnostic accuracy. Future prospective studies using standardized methodologies and uniform reference standards are required to allow meaningful comparisons. Additionally, sonication should be considered an adjunct diagnostic tool rather than a standalone test.

Some research groups contributed to multiple studies included in this review. Each publication was treated as a separate entry as variations in protocols were observed across studies with the same research groups. This approach may represent a risk of duplicate counting of the same research group. However, including the studies despite the same research groups was necessary to accurately map methodological variability. The review includes language restriction to English and German publications, omission of certain databases such as Web of Science, and a limited timeframe that does not cover the entire period since the introduction of sonication, with 2007 selected as the starting point due to the seminal study by Trampuz et al. (11).

### Conclusion

This scoping review revealed that published protocols for sonication in FRI and PJI show great heterogeneity across all methodological aspects. However, the lack of methodological standardization and the major variability between protocols hamper reproducibility and prevent the establishment of a reliable and universally accepted diagnostic threshold for clinical decision-making. Defining a consensus sonication protocol, including standardization of device settings, liquid composition, handling steps, and culture conditions, is, therefore, essential before diagnostic thresholds can be validated and implemented in routine practice.

## SUPPLEMENTAL MATERIAL

Supplemental material jcm.00495-26-s0001.docx Search strategy and PRISM files.

ASM does not own the copyrights to Supplemental Material that may be linked to, or accessed through, an article. The authors have granted ASM a non-exclusive, world-wide license to publish the Supplemental Material files. Please contact the corresponding author directly for reuse.
